# Thermal Analysis and Crystal Structure of Poly(Acrylonitrile-Co-Itaconic Acid) Copolymers Synthesized in Water

**DOI:** 10.3390/polym12010221

**Published:** 2020-01-16

**Authors:** Hailong Zhang, Ling Quan, Aijun Gao, Yuping Tong, Fengjun Shi, Lianghua Xu

**Affiliations:** 1 School of Materials Science and Engineering, North China University of Water Resources and Electric Power, Zhengzhou 450045, China; tongyuping@ncwu.edu.cn (Y.T.); shifengjun1962@126.com (F.S.); 2 Key Laboratory of Carbon Fiber and Functional Polymers Ministry of Education, Beijing University of Chemical Technology, Beijing 100029, China; bhgaoaijun@163.com (A.G.); xulh@mail.buct.edu.cn (L.X.); 3 School of Electric Power, North China University of Water Resources and Electric Power, Zhengzhou 450045, China; quanling@ncwu.edu.cn

**Keywords:** copolymers, morphology, crystallization, differential scanning calorimetry, thermal properties

## Abstract

The composition and structure of polyacrylonitrile (PAN) precursors play an important role during thermal stabilization, which influences the properties of the resulting carbon fibers. In this paper, PAN homopolymer and PAN-itaconic (IA) copolymers with different IA contents were synthesized by aqueous phase precipitation polymerization. The effects of IA content on the structure and thermal properties were studied using scanning electron microscopy (SEM), Fourier transform infrared spectroscopy (FTIR), X-ray diffraction (XRD), differential scanning calorimetry (DSC), and thermogravimetric analysis (TGA). The morphology of PAN polymers showed that the average size of the PAN particles increased with the increase of IA content in the feed. The content of the IA comonomer on the copolymers was quantitatively characterized by the relative absorbance intensity (A_1735_/A_2243_) in FTIR spectrum. With the increase of IA content in the feed, PAN-IA copolymers exhibited lower degree of crystallinity and crystal size than the control PAN homopolymer. The results from DSC curves indicated that PAN-IA1.0 copolymers had lower initial exothermic temperature (192.4 °C) and velocity of evolving heat (6.33 J g^−1^ °C^−1^) in comparison with PAN homopolymer (*T*_i_ = 238.1 °C and Δ*H*/Δ*T* = 34.6 J g^−1^ °C^−1^) in an air atmosphere. TGA results suggested that PAN-IA1.0 copolymers had higher thermal stability than PAN homopolymer, which can form a ladder structure easier during thermal processing. Therefore, PAN-IA1.0 copolymers would be a suitable candidate for preparing high performance PAN based carbon fibers.

## 1. Introduction

Carbon fibers have gained tremendous attention because of their excellent mechanical properties and low density, and can be used in the industrial field, aerospace, defense areas, and civil engineering [1,2,3]. Its precursor materials, such as polyacrylonitrile (PAN), pitch and rayon, need to undergo a series of processes to be converted into carbon fibers, including drawing and heat treatment [4,5,6]. Among the obtained carbon fibers, PAN based carbon fibers serve nearly 90% of the commercial carbon fiber market because of its low cost and high strength [7]. However, the PAN homopolymer is difficult to be used as carbon fiber precursors because of its poor drawability and heat stabilization caused by the extensive interaction of adjacent nitrile groups, which make the final carbon fibers of poor quality [8,9,10]. Therefore, a small amount of comonomers, such as itaconic acid (IA), and methacrylic acid (MA), have been used to enhance drawability and hydrophilicity of PAN copolymers, and especially to improve the heat stabilization and smooth the rate of heat release in order to obtain high performance carbon fibers [11,12,13]. In 2019, Liao et al. used the bifunctional poly(ethylene glycol) bisazide (PEG-BA) as an interconnecting molecule and obtained high strength and high toughness PAN precursor fibers, and this paper was published in Science [14]. Fox regarded that the PAN fibers produced by Liao et al. [14] had properties approaching those of dragline spider silk, and analyzed some strategies to create a new generation of high-performance carbon fibers [15].

PAN copolymers can be synthesized by different polymerization methods, including solution polymerization, aqueous phase precipitation polymerization, and mixed solvent deposited polymerization. The polymerization procedure affects the thermal behaviors of PAN copolymers [16,17]. For instance, PAN copolymers synthesized by solution polymerization showed lower maximum exothermic temperature than PAN copolymers prepared by aqueous phase precipitation polymerization, which could be ascribed to the differences in tacticity, molecular defects, molecular weight, and dispersity of the polymerization [18]. However, solution polymerization needs a long time of more than 24 h to achieve a high level of reaction conversion compared to 1–2 h of aqueous phase precipitation polymerization [19]. Therefore, aqueous phase precipitation polymerization is regarded as one of the most effective ways to produce copolymers in a large scale. Moreover, owing to the zero chain transfer coefficient of water, aqueous phase precipitation polymerization could provide PAN copolymers with high molecular weight, which is important for preparing high performance carbon fibers [20,21]. Furthermore, an initiator without metal ions must be chosen instead of the traditional initiator [19] because the metal ions would reduce the mechanical properties of carbon fibers.

Thermal stabilization of PAN copolymers at a temperature of about 180–400 °C is recognized as one of the most important stages for preparing carbon fibers [22]. Three main chemical reactions take place during this process, including cyclization, dehydrogenation, and oxidation [23]. The cyclization reaction includes the transformation of the linear molecular chain of PAN copolymers in a ladder structure, which makes them infusible and heat resistance. These ladder structures are conducive to the evolution of the turbostratic graphite-like structures at a carbonization stage with a higher temperature and affect the final quality of the resulting carbon fibers [24]. However, the cyclization reaction of PAN homopolymer happens at a relatively high temperature complying with a free radical mechanism, which is difficult to control because of the release of a huge amount of heat [25]. The incorporation of IA monomer into the PAN matrix initiated the cyclization reaction at a relatively low temperature through an ionic mechanism and relaxed the heat release [26,27,28]. Thus, many researchers studied the effects of IA on the thermal stabilization of PAN copolymers, which showed that the introduction of IA comonomer promoted cyclization reactions and broadened the range of exothermic temperature, and PAN-IA copolymers had lower activation energy of cyclization reaction during this processing [29,30,31,32,33,34,35]. Because the thermal stabilization depends on the nature and the content of comonomer as well as the polymerization methods of PAN-IA copolymers [9,11,19,30,32,35,36,37,38]. In the previous literature, the water soluble initiators were adopted, such as ammonium persulfate [9,30], potassium persulfate [9,21], or the mixture of sodium bisulfite and potassium persulfate [11]. We used ascorbic acid as initiator that did not contain other elements other than those contained in PAN-IA copolymers. Meanwhile, we studied the effects of IA content on the particle morphology and crystal structure for PAN-IA copolymers prepared by aqueous phase precipitation polymerization. The molar ratio of AN to IA was based on the long-time research experience of our groups for preparing PAN based carbon fibers.

In this work, PAN homopolymer and PAN-IA copolymers with different IA contents have been prepared by aqueous phase precipitation polymerization with the free radical polymerization mechanism. Ascorbic acid was used as the water soluble initiator. The effects of IA content on the morphology and crystalline structure of PAN were characterized by scanning electron microscopy (SEM) and X-ray diffraction (XRD), respectively. The chemical structure of PAN-IA copolymers were investigated through Fourier transform infrared spectra (FTIR), and the content of IA in PAN-IA copolymers was quantitatively characterized compared with the ratio of AN and IA in the feed. The thermal behavior of PAN homopolymer and PAN-IA copolymers were evaluated by differential scanning calorimetry (DSC) in a nitrogen and air atmosphere, and thermal stability was studied by thermogravimetric analysis (TGA) in a nitrogen atmosphere.

## 2. Materials and Methods

### 2.1. Materials

Acrylonitrile (AN) with analytical grade was purchased from the Sinopharm Chemical Reagent Co. Ltd. (Shanghai, China) and purified by distillation at the temperature about 77 °C to remove the inhibitors. Itaconic acid and ascorbic acid with analytical grade were also provided by the Sinopharm Chemical Reagent Co. Ltd. (Shanghai, China), which were used without further purification. Deionized water was used as the solvent.

### 2.2. Synthesis of PAN Homopolymer and PAN-IA Copolymers

**Figure 1:**
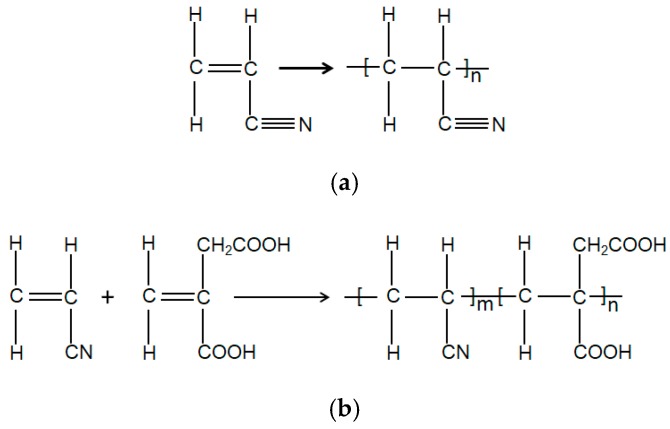
Synthetic route of (**a**) Polyacrylonitrile (PAN) homopolymer and (**b**) PAN-itaconic acid (IA) copolymers.

PAN-IA copolymers were synthesized by the free radical aqueous phase precipitation polymerization using ascorbic acid as the water soluble initiator. Deionized water (120 mL) was charged in a three neck round bottom flask equipped with a stir bar, and the flask was kept in a water bath at 60 °C. The solution was degassed with nitrogen to remove oxygen for 10 min. Then, 10.42 mL AN monomer was added into the flask while stirring depending on its solubility in deionized water, and the weighed IA comonomer with the molar ratio 99.5:0.5 (AN:IA) was mixed into the three necked flask with stirring for 5 min. In addition, 55.8 mg of ascorbic acid was added into the flask to initiate the reaction. The polymerization reaction was continued at 60 °C for 2 h in a nitrogen atmosphere, and then was cooled to room temperature. The mixture was filtered through 2.0 μm polytetrafluoroethylene membrane in vacuum and washed several times with deionized water to remove the unreacted monomers. Finally, the copolymers were dried in a vacuum oven at 45 °C for 12 h. The PAN-IA0.5 copolymers were obtained. PAN-IA1.0 copolymers with the molar ratio 99.0:1.0 (AN:IA) and PAN homopolymer were also polymerized in the same conditions. The synthesis reaction of the polymers is shown in Figure 1.

### 2.3. Characterization

Fourier transform infrared spectroscopy (Nicolet 5700 spectrometer, Thermo Nicolet Company, Madison, WI, USA) of PAN homopolymer and PAN-IA copolymers was used to detect the structural changes in the range of 4000–400 cm^−1^. The polymer powders were pressed into a pellet with KBr at the same mass, and 32 scans were collected at a resolution of 4 cm^−1^. In order to conduct quantitative analysis, the weight of the samples in each KBr disc was also the same. The content of IA units on the copolymer macromolecular chains was determined using the absorbance ratio of the –COOH groups and –CN groups. Scanning electron microscopy (Hitachi S 4700, Tokyo, Japan) was used to observe the morphology of the polymer at an accelerating voltage of 20 kV. The powder samples were sprayed with a thin platinum layer by ion beam coater using Q150R Plus (Quorum Technologies, East Sussex, UK). The exothermic properties of PAN polymer were studied by a differential scanning calorimeter with a Q 100 instrument (TA Company, Boston, MA, USA). The samples of about 3.0 mg were scanned using the DSC instrument from 50 to 400 °C at a heating rate of 10 °C min^−1^ in a nitrogen atmosphere and air atmosphere, respectively. Thermal stability study was carried out with a Netzsch TG 209 thermal gravimetric analyzer (Netzsch Group, Serb, Germany). About 10.0 mg of each polymer was heated from 50 °C to 800 °C at a heating rate of 20 °C min^−1^ in a nitrogen atmosphere with flow rate 100 mL min^−1^. X-ray diffraction patterns of PAN homopolymer and PAN-IA copolymers were obtained using a D/max 2500VB2+/PC diffractometer (Rigaku Corporation, Tokyo, Japan) with Cu Kα radiation at 30 kV and 20 mA, and scanning range from 5 to 55° with scan rate of 10° min^−1^ and a step of 1.0 s. All the measurements of powder samples were carried out in the same conditions. XRD parameters were calculated after the XRD profiles with Lorentzian cross product curves. The average crystal size was calculated from the XRD data by the Scherrer equation:

(1) $$L_{C}=\frac{Κ\lambda }{\beta cοs\theta },$$

where K is a constant that was taken to be 0.89; λ = 0.154056 nm is the wavelength of the Cu Kα X-rays; θ is the Bragg angle; β is the full width at half maximum intensity of the peak at about 2θ ≈ 16.8°; the strongest peak at about 16.8° is assigned to the (100) crystal plane of hexagonal lattice for the PAN polymer [39].

The degree of crystallinity (C%) was determined by the integral area of the crystalline zone and the amorphous zone according to the following formula:

(2) $$C(\%)=\frac{A_{c}}{A_{a}+A_{c}},$$

where $A_{c}$ is the integral area of the crystalline zone at around 2θ ≈ 16.8° in the XRD patterns; $A_{a}$ is the integral area of the amorphous zone in the XRD patterns [40].

## 3. Results and Discussion

### 3.1. Morphology of the PAN Polymer

**Figure 2:**
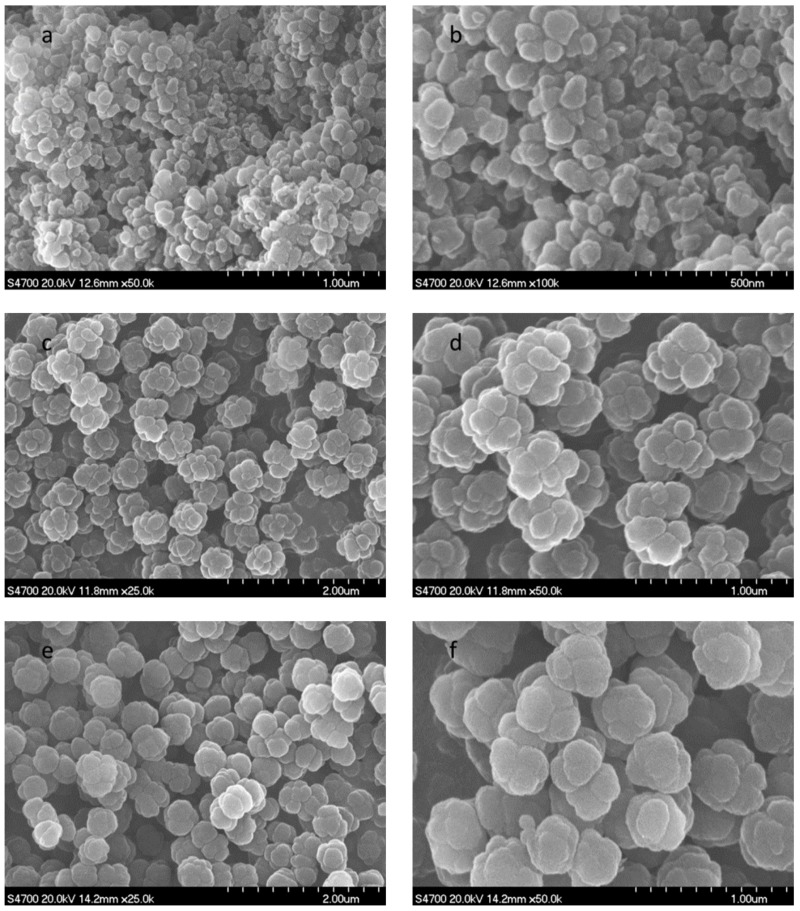
Scanning Electron Microscopy (SEM) micrographs of (**a**,**b**) PAN homopolymer, (**c**,**d**) PAN-IA0.5 copolymers, and (**e**,**f**) PAN-IA1.0 copolymers.

The effects of IA comonomer on the morphology of the PAN based copolymers are shown in Figure 2. PAN homopolymer obtained through the aqueous phase precipitation polymerization, as shown in Figure 2a,b, exhibits a large, irregular spherical shape with diameter of about 30–60 nm. With increasing the molar content of IA comonomer from 0.5 to 1.0, the average particle size of PAN copolymers increases from 120–170 nm to 200–250 nm, and the small particles aggregated to form large particles with diameter of about 400–500 nm. This may be ascribed to increasing of the polymerization rate with the increase of the comonomer concentration that can result in longer macromolecular chains [41]. The longer molecular chain length, the higher molecular weight. The long molecular chain is beneficial to obtain high tensile orientation in the process of stretching so as to improve the mechanical properties of PAN precursor fibers. The high molecular weight plays an important role in producing the high performance carbon fibers [19]. Moreover, PAN-IA1.0 copolymers have narrower particle size distribution and a more regular shape compared to the PAN homopolymer. These results indicate that the relative content of IA comonomer could change the morphology of PAN, which might affect the crystal structure owing to decrease of the adjacent interaction of nitrile groups [40]. On the one hand, the weak interaction force between the adjacent nitrile groups is beneficial to obtain a high drafting ratio for PAN precursor fibers. On the other hand, it more easily controlled the releasing of huge heat during the thermal stabilization.

### 3.2. FTIR Analysis

**Figure 3:**
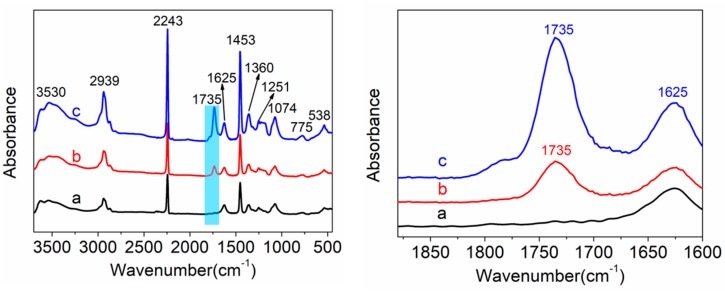
Fourier Transform Infrared Spectroscopy (FTIR) spectra of (**a**) PAN homopolymer, (**b**) PAN-IA0.5 copolymers, and (**c**) PAN-IA1.0 copolymers (**left** figure). Amplification section of –COOH groups in FTIR (**right** figure).

The FTIR spectra of a PAN homopolymer, as well as PAN-IA0.5 copolymers and PAN-IA1.0 copolymers, are shown in Figure 3. For the PAN homopolymer, a sharp and strong intensity of the absorption peak at 2243 cm^−1^ is ascribed to the –C≡N stretching vibration. The peak at about 3530 cm^−1^ is attributed to the –OH stretching vibration, the intensity of which becomes stronger with the increase of IA content in the feed, which is assigned to the –OH groups and the –CONH_2_ groups of the hydrolyzed PAN homopolymer [19]. The peaks at around 2939 cm^−1^ and 1453 cm^−1^ are assigned to the stretching vibration and the bending vibration of –CH_2_ groups, respectively [42]. The weak peak at about 1625 cm^−1^ is attributed to the hydrolysis of AN units during polymerization [43]. The peaks at around 1360 cm^−1^ and 1074 cm^−1^ are attributed to the symmetric bending vibration of –CH_3_ groups and the bending vibration of –CN groups. The peaks at around 775 cm^−1^ and 538 cm^−1^ are assigned to the out of plane bending of –C=CH groups and the twisting vibration of the –C=O groups, respectively. However, PAN-IA copolymers exhibit a new absorption peak at about 1735 cm^−1^ compared to PAN homopolymer, which is assigned to the stretching vibration of –COOH groups, and the relative intensity of this peak becomes stronger with increasing of IA content. This peak is used to quantify the content of –COOH groups on PAN macromolecular chains [44]. According to Lambert–Beer’s law, the ratio of the absorbance of the peak at 1735 cm^−1^ and 2243 cm^−1^ (A_1735_/A_2243_) is proportional to the ratio of the content IA and AN on the PAN macromolecular chains. The value of absorbance of these two peaks and the ratio of them is listed in Table 1.

From Table 1, we can see that the A_1735_/A_2243_ values of PAN-IA 0.5 and PAN-IA1.0 copolymers are 0.1589 and 0.3143, respectively, which is proportional to the increase of IA content in the feed. However, the ratio value of the IA content in copolymers is smaller than that of the IA content in the feed. The reason is that the polymerization rate of AN monomer and IA comonomer is inconsistent, and the proportion of IA monomer and AN comonomer in the feed changes as the reaction proceeds. Another reason is that IA comonomer could hinder the copolymerization reaction, and the unreacted IA or the generated IA oligomer may remain in the copolymer. Meanwhile, the proportional relationship of IA monomer and IA comonomer between in the feed and copolymers indicated that the reaction competition rate of AN and IA comonomer has not been significantly affected in these ratios, which were also confirmed by the results of FTIR spectroscopy [44], where PAN-IA copolymers were synthesized by an aqueous free radical polymerization.

### 3.3. XRD Analysis

**Figure 4:**
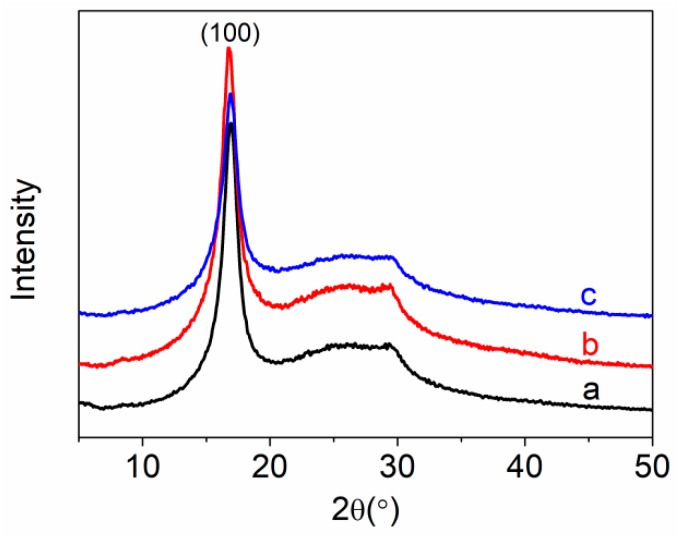
X-ray Diffraction (XRD) patterns of (**a**) PAN homopolymer, (**b**) PAN-IA0.5 copolymers, and (**c**) PAN-IA1.0 copolymers.

XRD patterns of PAN homopolymer and PAN-IA copolymers are shown in Figure 4. PAN homopolymer, as shown in Figure 4, shows the strongest diffraction peak at about 2θ ≈ 16.8°, corresponding to the (100) crystal plane of the hexagonal lattice with a stiff rod-like conformation owing to the intermolecular repulsion of the nitrile dipoles [45]. A weak diffraction peak at about 2θ ≈ 29.0° is assigned to the (101) crystal plane of hexagonal lattice [45]. The weak and broad peak at about 2θ ≈ 25.0° is attributed to the amorphous regions of PAN homopolymer. These results prove that PAN polymer is a semi-crystalline polymer. PAN-IA copolymers exhibit similar curves with a PAN homopolymer. However, the relative intensity of the peak at about 2θ ≈ 16.8° decreases with the increase of IA content in PAN-IA copolymers, suggesting that the IA comonomer in the PAN macromolecular chains blocks the intermolecular interactions between the nitrile groups [46]. The positions of the main peaks remain the same, indicating that the incorporation of IA comonomer did not destroy the crystal structure of the PAN homopolymer.

Table 2 shows the degree of crystallinity and crystal size of PAN homopolymer and PAN-IA copolymers according to Equations (1) and (2). The degree of crystallinity of PAN homopolymer is 35.05%, which is assigned to the (100) crystal plane of the hexagonal lattice for the nitrile groups. The crystal size of PAN homopolymer is 53.40 Å. However, the degree of crystallinity and the crystal size of PAN-IA0.5 copolymers are 28.98% and 50.46 Å, respectively, which is lower than that of the PAN homopolymer. When the molar ratio content of IA comonomer increases from 0.5 to 1.0, PAN-IA1.0 copolymers have a lower degree of crystallinity and crystal size than PAN-IA0.5 copolymers. These results are attributed to the ability of the IA comonomer to disrupt the regular macromolecular structure of the macromolecular chains of PAN [39].

### 3.4. Thermal Properties

**Figure 5:**
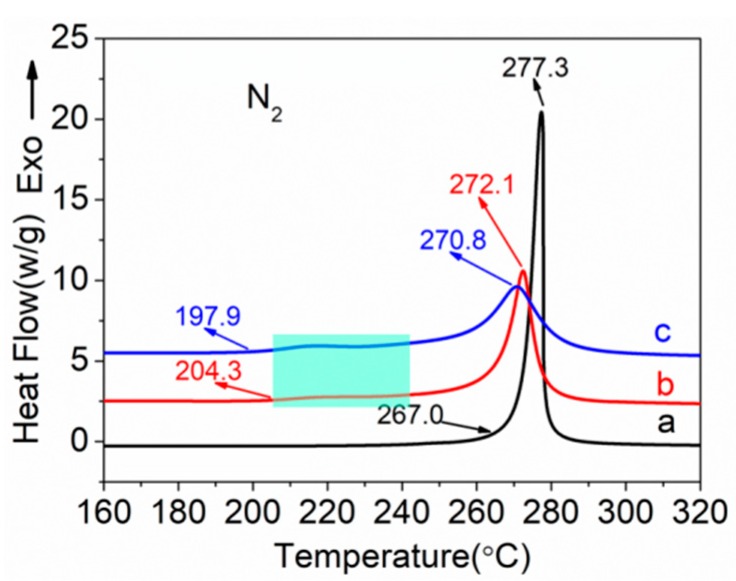
Differential Scanning Calorimetry (DSC) curves of (**a**) PAN homopolymer, (**b**) PAN-IA0.5 copolymers, and (**c**) PAN-IA1.0 copolymers in a nitrogen atmosphere.

DSC thermograms of PAN homopolymer and PAN-IA copolymers recorded in a nitrogen atmosphere at a rate of 10 °C min^−1^ are shown in Figure 5. PAN homopolymers underwent only a cyclization reaction of the nitrile groups with no other reactions occurring in a nitrogen atmosphere and formed a ladder structure, which released a lot of heat. Thus, it exhibited a sharp and narrow exothermic peak. The incorporation of IA into the PAN matrix significantly reduced the intensity of the exothermic peak of cyclization and shifted the maximum peak temperature to a lower temperature. Moreover, the copolymers exhibit an exothermic shift of the baseline, as shown in the shaded region in Figure 5. The various parameters obtained from the DSC curves, including the initial temperature (*T*_i_), the final temperature (*T*_f_), and their different temperature (Δ*T* = *T*_f_ − *T*_i_), the maximum peak temperature (*T*_m_), the evolved heat (Δ*H*), and the velocity of evolving heat (Δ*H*/Δ*T*) are summarized in Table 3.

**Figure 6:**
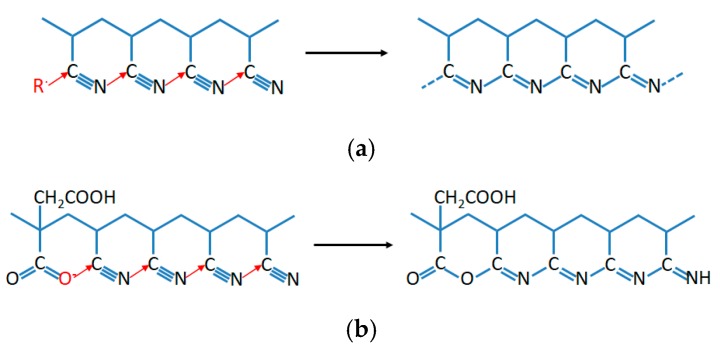
Cyclization reaction: (**a**) the free radical mechanism and (**b**) the ionic mechanism.

Table 3 shows the various parameters from the DSC curves of PAN homopolymer, PAN-IA0.5 copolymers and PAN-IA1.0 copolymers in a nitrogen atmosphere. The *T*_i_ and *T*_f_ of the PAN homopolymer are about 267.0 °C and 281.5 °C, respectively, and the differences between *T*_i_ and *T*_f_ is about 14.5 °C. The *T*_m_ of cyclization is about 277.3 °C and the evolved heat is about 567.1 J g^−1^. According to these exothermic parameters, the velocity of evolving heat is 39.1 J g^−1^ °C^−^^1^. The incorporation of IA comonomer significantly decreased the *T*_i_ of the exothermic peak. The *T*_i_ of PAN-IA0.5 copolymers and PAN-IA1.0 copolymers is about 204.3 °C and 197.9 °C, respectively, which is much lower than that of the PAN homopolymer. As we know, the PAN homopolymer underwent cyclization reaction in the absence of oxygen by the free radical mechanism (Figure 6a), which could be initiated at a relative higher temperature [18]. However, the IA comonomer with nucleophilic of –COOH groups is able to initiate the cyclization reaction at a lower temperature via an ionic mechanism (Figure 6b) [35]. With the increasing of IA content, the *T*_i_ of PAN copolymers decreases from 267.0 °C to 204.3 °C and 197.9 °C, suggesting that the incorporation of IA comonomer could change the mechanism of cyclization reaction of nitrile groups [35]. The *T*_m_ of PAN-IA0.5 copolymers and PAN-IA1.0 copolymers is about 272.1 °C and 270.8 °C, respectively, which is lower than PAN homopolymer at 277.3 °C. The *T*_f_ of PAN-IA copolymers increases with increasing the IA content in the feed. Therefore, PAN-IA copolymers with higher IA content have the broader exothermic temperature range (Δ*T*) and the lower rate of exothermic reaction (Δ*H*/Δ*T*), indicating that the exothermic cyclization reaction of the copolymers in a nitrogen atmosphere is more easily controlled as compared with the PAN homopolymer.

**Figure 7:**
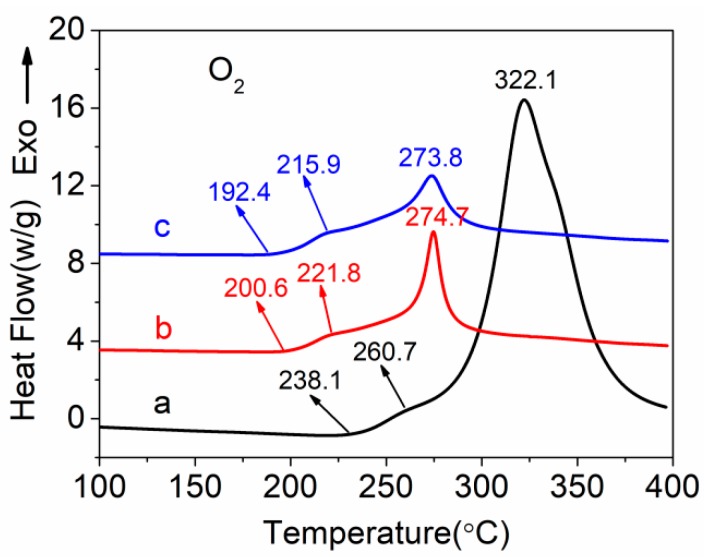
DSC curves of (**a**) PAN homopolymer, (**b**) PAN-IA0.5 copolymers, and (**c**) PAN-IA1.0 copolymers in an air atmosphere.

DSC thermograms of PAN homopolymer and PAN-IA copolymers with different IA contents measured in an air atmosphere at a rate of 10 °C min^−1^ are shown in Figure 7. PAN homopolymer exhibits two exothermic peaks in air in contrast with one in nitrogen. The weak shoulder peak at about 260.7 °C for PAN homopolymer is assigned to the cyclization reaction, and the strong exothermic peak at about 322.1 °C is ascribed to the oxidative reaction owing to oxygen in air. Meanwhile, the intensity of exothermic peak of PAN homopolymer in an air atmosphere decreases and the range of exothermic temperature (Δ*T*) becomes broad compared with PAN homopolymer in a nitrogen atmosphere. With increasing the content of the IA comonomer, the intensity of exothermic peak for PAN-IA copolymers decreases and the exothermic reaction starts at a much lower temperature. The relevant parameters of the exothermic curves, including the initial temperature (*T*_i_), the final temperature (*T*_f_), and their different temperature (Δ*T* = *T*_f_ − *T*_i_), the maximum peak temperature (*T*_m1_ and *T*_m2_), the evolved heat (Δ*H*), and the velocity of evolving heat (Δ*H*/Δ*T*) are listed in Table 4.

From Table 4, we can see that the *T*_i_ of PAN hompolymer in an air atmosphere is 238.1 °C, which is lower than the PAN homopolymer with 267.0 °C in a nitrogen atmosphere. This is ascribed to elemental oxygen in the air inducing the exothermic reaction. The *T*_f_ of PAN hompolymer is 375.2 °C. Thus, PAN homopolymer has a much wider range of exothermic temperature (Δ*T*) in an air atmosphere than the nitrogen atmosphere. The Δ*H* of PAN homopolymer also increases from 567.1 J g^−1^ in a nitrogen atmosphere to 4738 J g^−1^ in an air atmosphere. Then, it was observed that PAN homopolymer had a lower heating release rate (34.6 J g^−1^ °C^−1^) in an air atmosphere compared with the value of 39.1 J g^−1^ °C^−1^ in a nitrogen atmosphere. However, the *T*_i_ of PAN-IA0.5 copolymers and PAN-IA1.0 copolymers is 200.6 and 192.4 °C, respectively, which are lower than that of the PAN homopolymer. This suggests that the IA comonomer plays an important role in inducing the exothermic reaction. The *T*_m1_ and *T*_m2_ of PAN-IA copolymers also shift to a lower temperature with increasing the IA content. In addition, the incorporation of IA comonomer significantly decreases the value of Δ*H* of PAN homopolymer. As the molar ratio of IA increases from 0.5 to 1.0, the value of Δ*H* decreases from 1112 J g^−1^ to 1090 J g^−1^, respectively, which are both lower than that of the PAN homopolymer (4738 J g^−1^). Furthermore, the incorporation of IA comonomer decreases the Δ*H*/Δ*T* from 34.6 J g^−^^1^ °C^−^^1^ for PAN homopolymer to 6.64J g^−^^1^ °C^−^^1^ for PAN-IA0.5, 6.33 J g^−^^1^ °C^−^^1^ for PAN-IA1.0, respectively, indicating that PAN-IA copolymers have a lower exothermic rate than PAN homopolymer in the exothermic reaction, which is favorable to control the behavior of PAN polymers during thermal stabilization.

### 3.5. Thermal Stability

**Figure 8:**
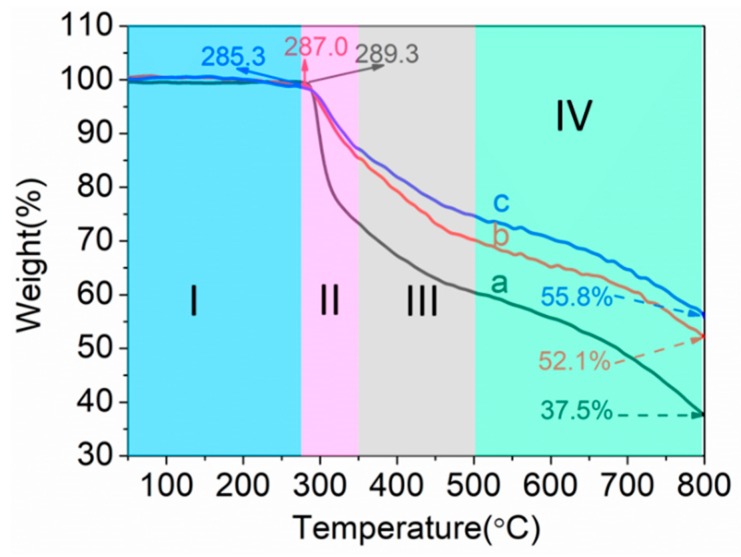
Thermogravimetric Analysis (TGA) curves of (**a**) PAN homopolymer, (**b**) PAN-IA0.5 copolymers, and (**c**) PAN-IA1.0 copolymers in a nitrogen atmosphere.

TGA was used to evaluate the thermal stability of PAN homopolymer, PAN-IA0.5 copolymers, and PAN-IA1.0 copolymers. The TGA curves in a nitrogen atmosphere with a heating rate of 20 °C min^−1^ are shown in Figure 8, which can be divided into four stages according to the weight loss in different temperature ranges. The TGA parameters obtained from the TGA curves about the initial decomposition temperature and the residual weight at different temperatures are listed in Table 5.

In the first region (I), PAN has slight weight loss owing to the water molecule and low molecular weight oligomers. The residual weights at 285 °C are listed in Table 5. PAN hompolymer has slightly higher residual weight than PAN-IA copolymers. In the second region (II), the initial decomposition temperature of PAN homopolymer is 289.3 °C, which is higher than that of PAN-IA0.5 with 287.0 °C and PAN-IA1.0 with 285.3 °C. This is consistent with the results from the DSC analysis in a nitrogen atmosphere, and indicating that the copolymers have easily undergone cyclization reaction compared to PAN homopolymer. PAN homopolymer shows an abrupt weight loss up to 350 °C, leaving residual weight about 73.3%. However, the residual weight of PAN-IA0.5 and PAN-IA1.0 copolymers at 350 °C is 85.4% and 87.2%, respectively. This suggests that the IA comonomer promotes the cyclization reaction at a lower temperature, and impedes the weight loss of PAN, and enhances the formation of the ladder structure through the ionic reaction mechanism [13]. In the third stage (III), PAN homopolymer and PAN-IA0.5 copolymers have a similar weight loss rate, and PAN-IA1.0 copolymers have a smaller weight loss compared with the other two. In the fourth stage (IV), the weight loss rate basically remains the same for the three samples. A PAN homopolymer has a residual weight of 37.5% at 800 °C, and PAN-IA0.5 with 52.1% and PAN-IA1.0 with 55.8%. These results suggest that the incorporation of IA comonomer changed the mechanism of cyclization reaction for nitrile groups and promoted the conversion of from ladder structures to turbostratic graphite-like structures during the lower carbonization stage [19].

**Figure 9:**
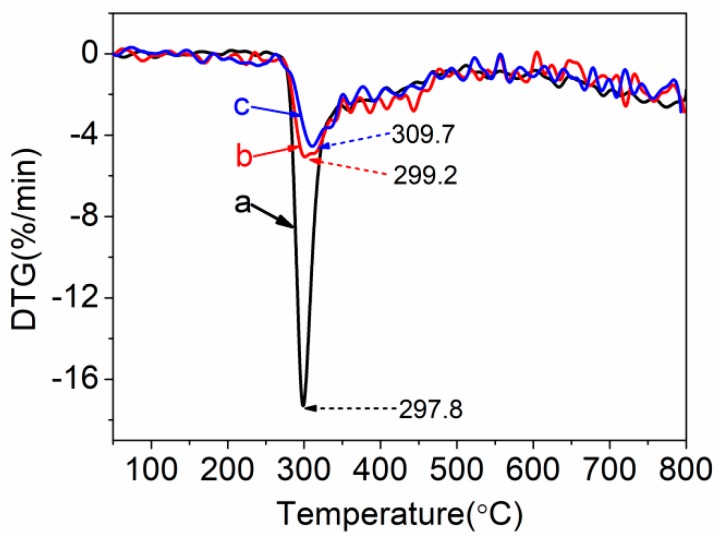
Derivative Thermogravimetric (DTG) curves of (**a**) PAN homopolymer, (**b**) PAN-IA0.5 copolymers and (**c**) PAN-IA1.0 copolymers.

The derivative thermogravimetric (DTG) curves of PAN, PAN-IA0.5, and PAN-IA1.0 are shown in Figure 9. The rate of weight loss for PAN homopolymer was faster than that of PAN copolymers. The temperature of the maximum rate of weight loss for PAN homopolymer is 297.8 °C, which is lower than that of PAN-IA0.5 at 299.2 °C and PAN-IA1.0 at 309.7 °C. This indicated that PAN copolymers could form more of the ladder structure than the PAN homopolymer, and suggested that the ionic mechanism of cyclization reaction had promoted the formation of a more perfect ladder structure than the free radical mechanism [13].

## 4. Conclusions

In summary, PAN homopolymer, PAN-IA0.5, and PAN-IA1.0 copolymers were synthesized by aqueous phase precipitation polymerization under the same conditions. The PAN homopolymer exhibited an irregular spherical morphology with a diameter of about 30–60 nm. With increasing the IA content in the feed, PAN-IA copolymers showed a larger diameter and more regular morphology compared to the PAN homopolymer. A new peak at about 1735 cm^−1^ in the FTIR spectrum indicated that PAN-IA copolymers contained the IA units in the macromolecular chains, and the content of IA units in the copolymers was proportional to the molar ratio of IA and AN in the feed, according to the relative value of the absorbance intensity of the two peaks at about 1735 cm^−1^ and 2243 cm^−1^. The degree of crystallinity and crystal size of PAN homopolymer was 35.05% and 53.40 Å, respectively, which were higher than that of PAN-IA0.5 with 28.98% and 50.46 Å, as well as PAN-IA1.0 with 27.17% and 49.17 Å. The incorporation of IA comonomer significantly decreased the initial temperature of stabilization in a nitrogen atmosphere (about 63–69 °C) than in an air atmosphere (about 38–45 °C). However, the maximum temperature of PAN-IA copolymers was lower than that of the PAN homopolymer in a nitrogen atmosphere, and even lower in an air atmosphere. This reduction could be ascribed to the cyclization reaction proceeding with an ionic mechanism in PAN-IA copolymers, owing to the nucleophilic of COOH groups on the IA unit, while following a free radical mechanism in PAN homopolymer. DSC data also exhibited that PAN-IA copolymers had a broader range of exothermic temperature and lower velocity of evolving heat in a nitrogen atmosphere as well as in an air atmosphere. The TGA results showed that, with increasing the IA content, the residual weight of PAN-IA comopolymers was higher at the same temperature, and the rate of weight loss at about 280–325 °C was lower. In other words, PAN-IA1.0 copolymers have a wider range of exothermic temperature, lower initial temperature, and velocity of evolving heat than PAN-IA0.5 copolymers, which is more suitable for preparing high performance PAN based carbon fibers.

## Figures and Tables

**Table 1:** The value of absorbance of the peak at the 2243 and 1735 cm^−1^.

Samples	The Value of Absorbance		A_1735_/A_2243_
	2243 cm^−1^	1735 cm^−1^	
Polyacrylonitrile (PAN)-IA0.5	0.2945	0.0468	0.1589
PAN-IA1.0	0.5450	0.1713	0.3143

**Table 2:** X-ray Diffraction (XRD) parameters of PAN homopolymer, PAN-IA0.5 copolymers, and PAN-IA1.0 copolymers.

Samples	Degree of Crystallinity (%)	Crystal Size (Å)
PAN	35.05	53.40
PAN-IA0.5	28.98	50.46
PAN-IA1.0	27.17	49.17

**Table 3:** Differential Scanning Calorimetry (DSC) parameters of PAN homopolymer and PAN-IA copolymers in a nitrogen atmosphere.

Samples-N_2_	*T*_i_ (°C)	*T*_f_ (°C)	*T*_m_ (°C)	Δ*H* (J g^−1^)	Δ*T* (°C)	Δ*H*/Δ*T* (J g^−1^ °C^−1^)
PAN	267.0	281.5	277.3	567.1	14.5	39.1
PAN-IA0.5	204.3	287.6	272.1	526.7	83.3	6.32
PAN-IA1.0	197.9	295.6	270.8	534.1	97.7	5.47

**Table 4:** DSC parameters of PAN homopolymer and PAN-IA copolymers in an air atmosphere.

Samples-O_2_	*T*_i_ (°C)	*T*_m1_ (°C)	*T*_m2_ (°C)	*T*_f_ (°C)	Δ*H* (J g^−1^)	Δ*T* (°C)	Δ*H*/Δ*T* (J g^−1^ °C^−1^)
PAN	238.1	260.7	322.1	375.2	4738	137.1	34.6
PAN-IA0.5	200.6	221.8	274.7	368.2	1112	167.6	6.64
PAN-IA1.0	192.4	215.9	273.8	364.6	1090	172.2	6.33

**Table 5:** Thermogravimetric Analysis (TGA) data of thermal stability for the different PAN polymers.

Samples	Initial Decomposition Temperature (°C)	Residual Weight at 285 °C (%)	Residual Weight at 350 °C (%)	Residual Weight at 500 °C (%)	Residual Weight at 800 °C (%)
PAN	289.3	99.1	73.3	60.4	37.5
PAN-IA0.5	287.0	98.7	85.4	70.2	52.1
PAN-IA1.0	285.3	98.2	87.2	74.7	55.8
